# Composite Epstein-Barr virus-positive mucosa-associated lymphoid tissue lymphoma and Epstein-Barr virus-negative diffuse large B-cell lymphoma in the parotid salivary gland of a patient with Sjögren’s syndrome and rheumatoid arthritis: a case report

**DOI:** 10.1186/s13256-019-2331-1

**Published:** 2020-01-17

**Authors:** Vadim R. Gorodetskiy, Natalya A. Probatova, Dmitry M. Konovalov, Natalya V. Ryzhikova, Yulia V. Sidorova, Andrey B. Sudarikov, Olga V. Mukhortova

**Affiliations:** 1 Department of Intensive Methods of Therapy, V.A. Nasonova Research Institute of Rheumatology, Kashirskoye shosse 34А, Moscow, 115522 Russia; 2 Department of Pathology, N.N. Blokhin Russian Cancer Research Center, Kashirskoye shosse 24, Moscow, 115478 Russia; 3 Department of Pathology, Dmitry Rogachev Research and Clinical Centre of Pediatric Hematology, Oncology and Immunology, Leninsky Prospect, 117, Moscow, 117513 Russia; 4 Laboratory of Molecular Hematology, National Hematology Research Center, Novy Zykovskiy Proezd, 4a, Moscow, 125167 Russia; 5 Department of Nuclear Diagnostics, A.N. Bakulev National Medical Research Center of Cardiovascular Surgery, Roublyevskoe Shosse 135, Moscow, 121552 Russia

**Keywords:** Composite lymphoma, Mucosa-associated lymphoid tissue lymphoma, Diffuse large B-cell lymphoma, Epstein-Barr virus, Autoimmune diseases

## Abstract

**Background:**

Epstein-Barr virus is associated with many human hematopoietic neoplasms; however, Epstein-Barr virus-positive mucosa-associated lymphoid tissue lymphoma is extremely rare. In routine clinical practice, detection of mucosa-associated lymphoid tissue lymphoma and diffuse large B-cell lymphoma in a tissue sample presumes a clonal relation between these neoplasms and that diffuse large B-cell lymphoma developed by transformation of the mucosa-associated lymphoid tissue lymphoma. However, evidence to support this presumption is sparse and controversial. Assessment of the clonal relationship of the lymphoid components of a composite lymphoma is important for understanding its pathogenesis and correct diagnosis.

**Case presentation:**

We present an unusual case of composite lymphoma (Epstein-Barr virus-positive mucosa-associated lymphoid tissue lymphoma/Epstein-Barr virus-negative diffuse large B-cell lymphoma) in the parotid salivary gland of a 62-year-old Caucasian woman with Sjögren’s syndrome and rheumatoid arthritis. Simultaneous occurrence of mucosa-associated lymphoid tissue lymphoma and diffuse large B-cell lymphoma in the parotid salivary gland led us to initially assume a clonal relationship between diffuse large B-cell lymphoma and mucosa-associated lymphoid tissue lymphoma. Epstein-Barr virus was detected by *in situ* hybridization and polymerase chain reaction in the mucosa-associated lymphoid tissue lymphoma, but not in diffuse large B-cell lymphoma, suggesting that these lymphomas were not clonally related. Fragment analysis of frame region 3 polymerase chain reaction products from microdissected mucosa-associated lymphoid tissue lymphoma and diffuse large B-cell lymphoma components revealed different clonal pattern rearrangements of the immunoglobulin heavy chain gene.

**Conclusions:**

Our patient’s case highlights the importance of assessing the clonal relationships of the lymphoid components of a composite lymphoma and Epstein-Barr virus screening in mucosa-associated lymphoid tissue lymphoma in patients with autoimmune disease.

## Introduction

A composite lymphoma is comprised of lymphomas with varying morphologies in the same topographic site at the time of presentation. However, a low-grade lymphoma that develops into a high-grade lymphoma is considered to be a lymphoma transformation, not a composite lymphoma [1]. Assessment of the clonal relationship of the lymphoid components of a composite lymphoma is important for understanding its pathogenesis and correct diagnosis. In routine clinical practice, detection of mucosa-associated lymphoid tissue (MALT) lymphoma and diffuse large B-cell lymphoma (DLBCL) in a tissue sample presumes a clonal relationship between these neoplasms and that DLBCL developed by transformation of the MALT lymphoma. However, evidence to support this presumption is sparse and controversial [2, 3].

Epstein-Barr virus (EBV) has been linked with many human hematopoietic neoplasms; however, MALT lymphoma does not belong to the group of EBV-associated lymphoproliferative disorders (LPDs) [4, 5], and EBV-positive MALT lymphoma is extremely rare. To date, to the best of our knowledge, only five cases of EBV-positive MALT lymphoma of the parotid gland have been described. Three cases of EBV-positive MALT lymphoma of the parotid glands were observed in patients with autoimmune pathology: in two cases of Sjögren’s syndrome (SS) [6] and in one case of a combination of rheumatoid arthritis (RA) and SS [7]. The remaining two cases of EBV-positive MALT lymphoma of the parotid gland had congenital immune deficiencies [7, 8]. However, composite lymphoma was not detected in any of the five cases, and the progression from low-grade to high-grade lymphoma was not suspected.

Here, we present an unusual case of composite lymphoma in the parotid salivary gland of a patient with SS and RA. The lymphoma was comprised of two clonally unrelated B-cell lymphomas: EBV-positive MALT lymphoma and EBV-negative DLBCL.

## Case presentation

In June 2012, a 62-year-old Caucasian woman was admitted to the V.A. Nasonova Research Institute of Rheumatology complaining of a rapid increase in the size of her left parotid gland starting 1 month prior to admission. A slight increase in the left parotid gland had been observed since 2007, with no clear upward trend; however, the cause of this increase was not determined. The patient’s medical history was consistent with a 19-year course of RA. Since 2002, she had complained of dryness in the eyes and mouth. Ophthalmologic examination revealed keratoconjunctivitis sicca, and dental examination revealed reduction of salivation to 3.00 ml. SS was then diagnosed. The patient was treated with gold therapy from 1998 to 2002. From 2002 to 2006, she was treated with methotrexate (MTX) at a dose of 15 mg/week. From 2006 to 2012, she was treated with leflunomide (Arava; Aventis Pharmaceuticals, Strasbourg, France).

At the time of admission, the patient’s physical examination showed a marked increase of the left parotid gland, subluxations of the metacarpophalangeal joints, and ulnar deviation of fingers. No night sweats, weight loss, or fever were found. Her peripheral blood counts, electrolytes, renal and liver function, and C-reactive protein were within normal limits. Her serum lactate dehydrogenase and β_2_-microglobulin levels were elevated to 273 IU/L (normal < 220) and 8.6 mg/L (normal < 3.0), respectively. Her anti-SSA/Ro, anti-SSB/La, anti-cyclic citrullinated peptide antibody, and antinuclear antibody levels were within normal ranges. Her rheumatoid factor level was 487 IU/ml (normal < 15), and her antibody titers against thyroglobulin and thyroid peroxidase were 183.3 U/ml (normal < 150) and 462.5 U/ml (normal < 75), respectively.

**Fig. 1:**
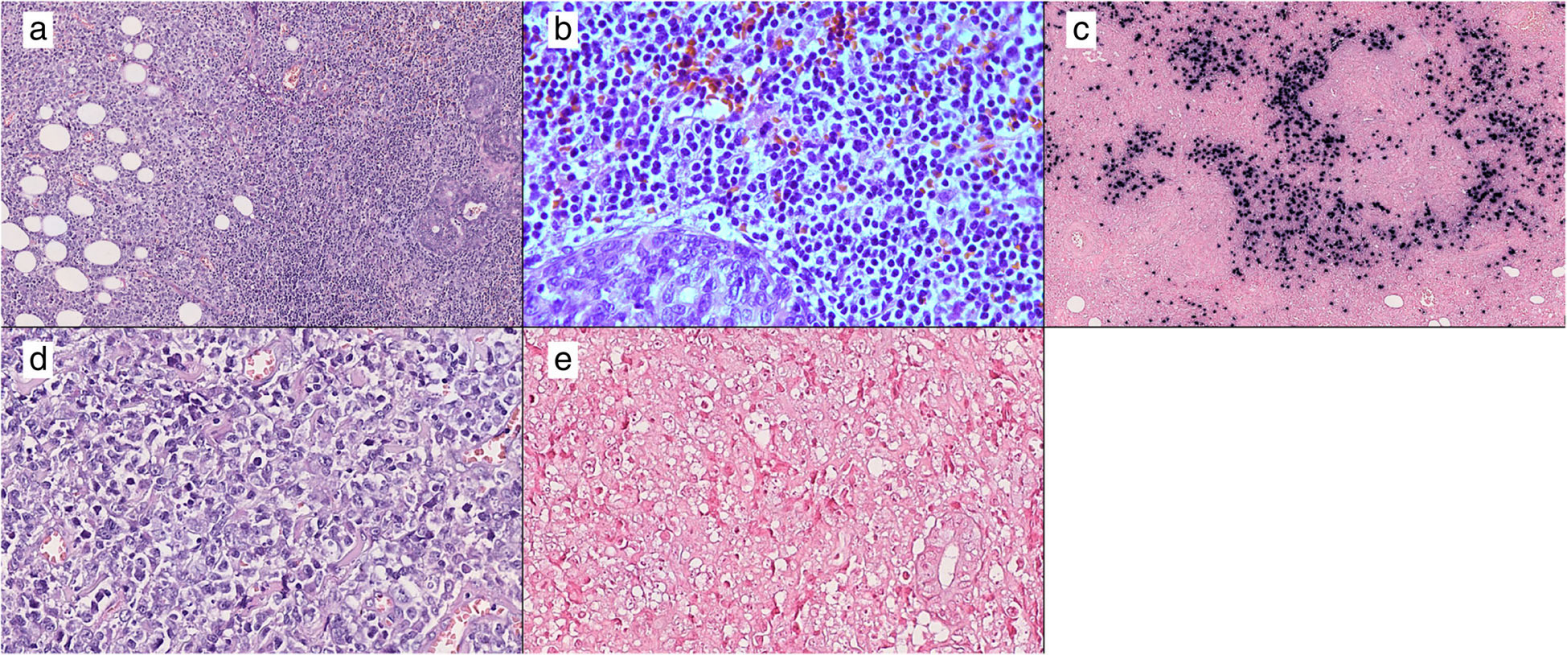
Parotid salivary gland. **a** Acinar atrophy due to diffuse infiltration of two distinct cell populations. Large lymphocytes are seen in left portion of the image, and the epithelial structures of the salivary gland with infiltration of small lymphocytes are seen in the right portion. H&E stain, 100× magnification. **b** The area of the parotid salivary gland affected by mucosa-associated lymphoid tissue (MALT) lymphoma. Small lymphocytes with round or centrocyte-like nuclei with moderately dispersed chromatin and inconspicuous nucleoli are seen. H&E stain, 400× magnification. **c** The area of the parotid salivary gland affected by MALT lymphoma. Epstein-Barr virus (EBV)-positive lymphocytes. EBV-encoded small ribonucleic acids (EBERs) detected by *in situ* hybridization (ISH), 100× magnification. **d** Area of the parotid salivary gland affected by diffuse large B-cell lymphoma (DLBCL). Large lymphocytes with oval to round and irregular nuclei with prominent nucleoli, with a moderate amount of cytoplasm. H&E stain, 400× magnification. **e** Area of the parotid salivary gland affected by DLBCL. EBV-negative large lymphocytes. EBERs detected by ISH, 400× magnification

Histologic examination of the enlarged parotid salivary gland revealed acinar atrophy due to diffuse infiltration by two distinct cell populations (Fig. 1 a). A portion of the salivary gland was effaced by small lymphocytes with round or irregular (centrocyte-like) nuclei with moderately dispersed chromatin and inconspicuous nucleoli (Fig. 1 b). The infiltrate contained large salivary gland ducts with epithelial proliferation and lymphoepithelial lesions. A few scattered medium-sized cells with light oval to round nuclei with nucleoli (resembling centroblasts) were observed in this area of the specimen. Neoplastic cells were positive for CD20, Bcl-2, and EBV-encoded small ribonucleic acids (EBERs) by *in situ* hybridization (ISH) (Fig. 1c) and were negative for Bcl-6, MUM1, HGAL, CD10, cyclin D1, CD30, and latent membrane protein 1 (LMP1). Ki-67 labeling showed a low (approximately 7%) proliferation index in this portion of the specimen. CD23 staining detected a destroyed meshwork of follicular dendritic cells (FDCs). Moreover, the infiltrate contained an abundance of reactive CD3^+^ and CD5^+^ T cells. Overall, this portion of the specimen was most consistent with EBV-positive MALT lymphoma. In the same specimen, but distinctly separate from the above lesion, there was a population of large lymphocytes with oval to round and irregular nuclei and prominent nucleoli, with a moderate amount of cytoplasm (Fig. 1d). These neoplastic cells were positive for CD20, Bcl-2, Bcl-6, MuM1, and HGAL and negative for CD10, cyclin D1, CD30, LMP1, and EBERs by ISH (Fig. 1e). The Ki-67 staining in this portion of the specimen was approximately 80% and lacked FDCs and lymphoepithelial lesions. This portion of the specimen was consistent with EBV-negative DLBCL of the nongerminal center of the B-cell subtype (according to the Hans algorithm) [9].

**Fig. 2:**
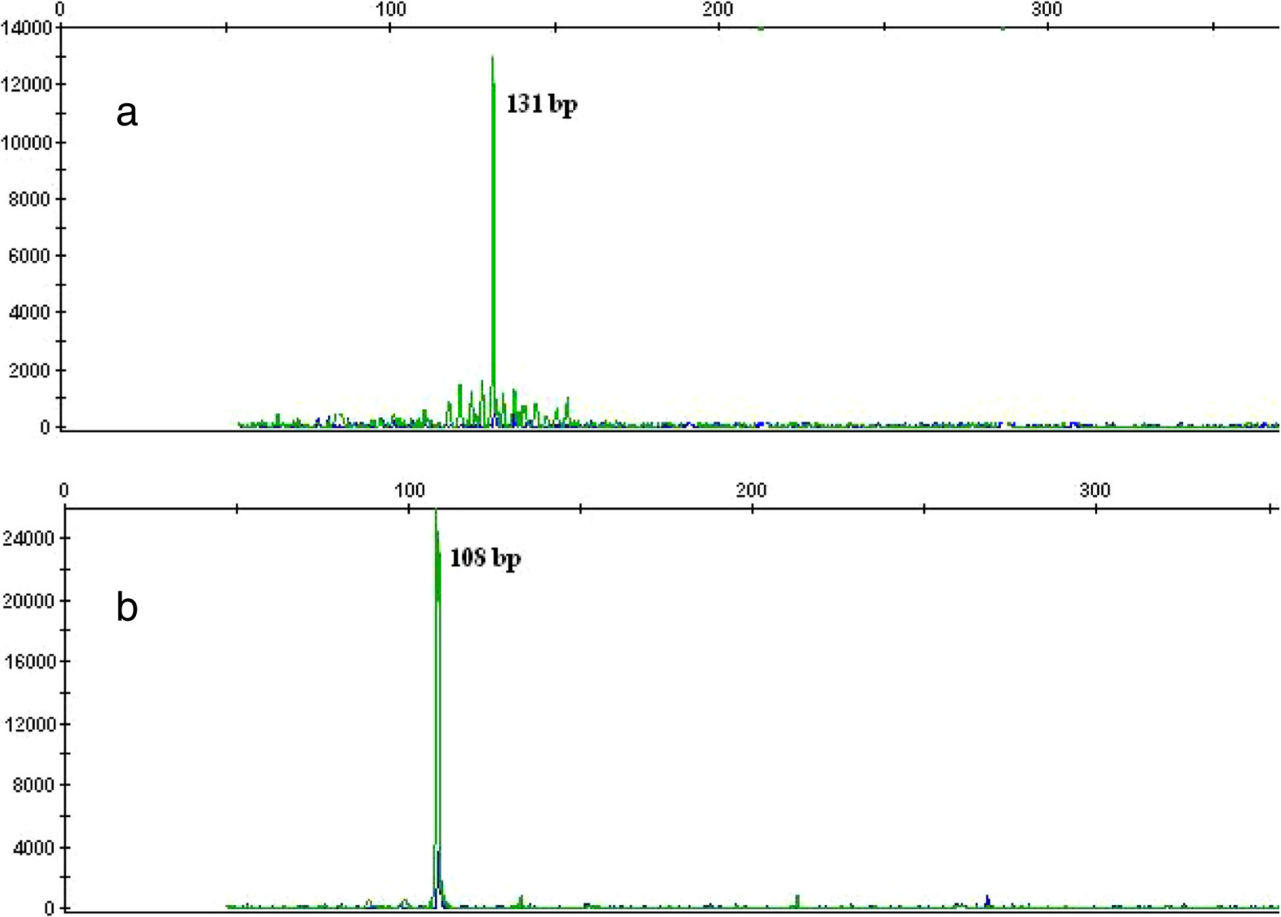
Fragment analysis of frame region 3 polymerase chain reaction products from the parotid salivary gland shows the different clonal pattern rearrangements of the immunoglobulin heavy chain gene between diffuse large B-cell lymphoma (DLBCL) and mucosa-associated lymphoid tissue (MALT) lymphoma. **a** Microdissected DLBCL component. **b** Microdissected MALT lymphoma component

To determine the clonal relationship between the large- and small-cell components, we microdissected the morphologically distinct tumor components. DNA was extracted from formalin-fixed, paraffin-embedded tissue blocks in samples of the MALT lymphoma and DLBCL. The BIOMED primer set and standardized protocol were used to study rearrangements of the immunoglobulin heavy chain (IGH) gene [10]. IGH frameworks 1, 2, and 3 assays (tube A, tube B, and tube C) were used to detect VH-JH rearrangements. The fragments were detected on an ABI PRISM 3130 Genetic Analyzer (Applied Biosystems, Foster City, CA, USA), and the data were analyzed with GeneMapper software version 4.0 (Applied Biosystems). Fragment analysis showed different clonal pattern rearrangements of the IGH gene between the MALT lymphoma and DLBCL (Fig. 2). We could not identify EBV DNA in the DLBCL-containing portion of the specimen by polymerase chain reaction (PCR), and breaks in the *MALT1*/18q21 gene in the MALT lymphoma-containing portion by fluorescence *in situ* hybridization.

**Fig. 3:**
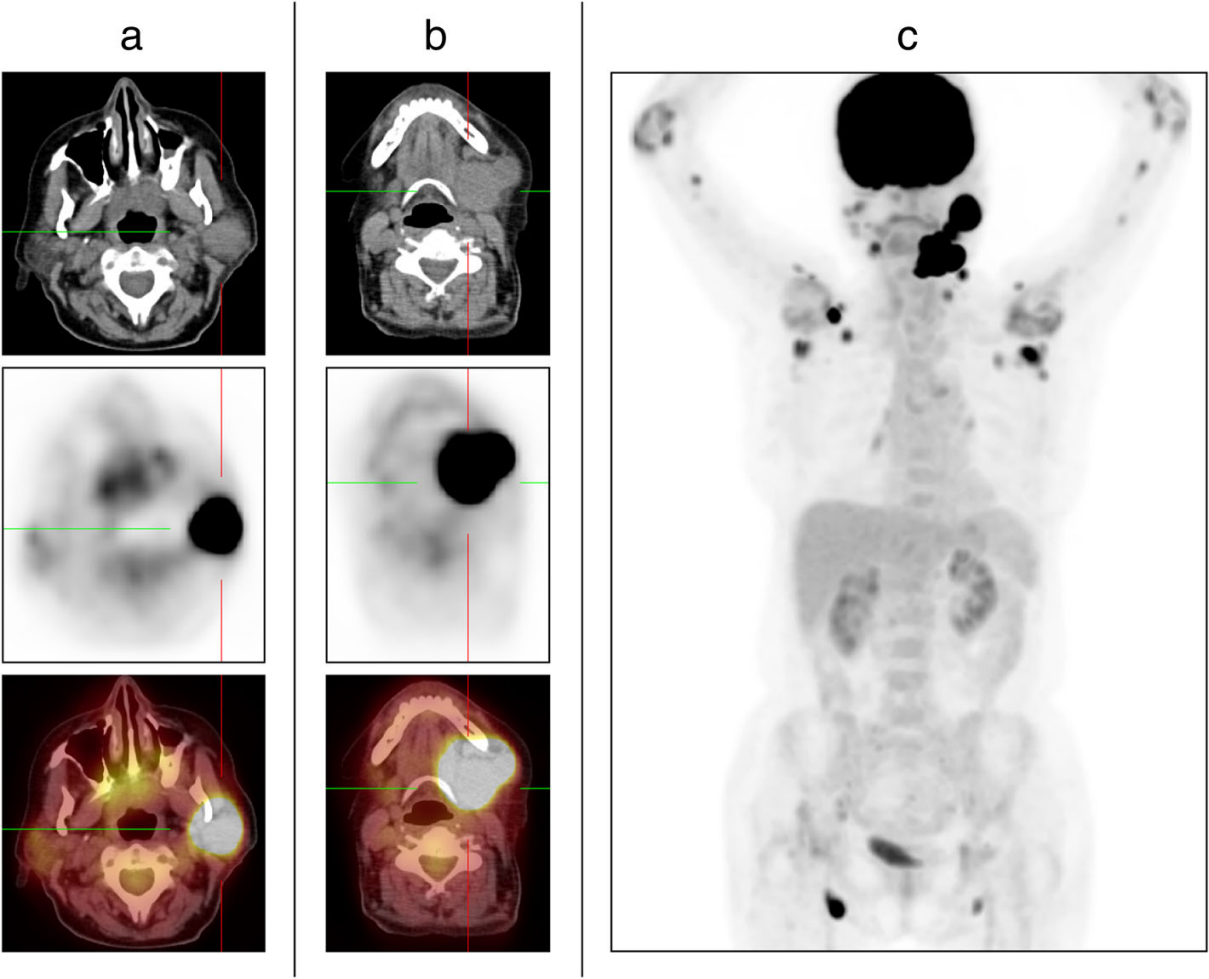
Fluorine-18 fluorodeoxyglucose (FDG) positron emission tomography combined with computed tomography. **a** Increased FDG accumulation in the left parotid gland. **b** Increased FDG accumulation in the left submandibular gland. **c** Multiple pathological foci of FDG uptake with maximum standardized uptake value up to 50.1

Fluorine-18 fluorodeoxyglucose (FDG) positron emission tomography combined with computed tomography revealed multiple foci of increased FDG accumulation merging into conglomerates in the left parotid (Fig. 3 a) and left submandibular (Fig. 3b) areas, with a maximum standardized uptake value (SUVmax) of up to 50.1. Multiple separated foci of increased FDG accumulation were found in the left cervical, right parotid, right mandibular, and right clavicular areas, as well as in the axillary and groin areas bilaterally (SUVmax 12.3). In addition, we identified individual small (up to 12 mm in diameter) foci of increased FDG accumulation in the soft tissues of the inner surface of the upper limbs along the humeral bones (Fig. 3c).

Leflunomide (Arava) was canceled in July 2012, and R-CHOP (rituximab, cyclophosphamide, doxorubicin, vincristine, and prednisone) immunochemotherapy was subsequently started. After six cycles, complete metabolic response was achieved and has been maintained in a follow-up period of 72 months. Rheumatoid factor and antibodies to thyroglobulin and thyroid peroxidase returned to normal levels following immunochemotherapy and stayed within normal limits during the entire follow-up period.

## Discussion

EBV is lymphotropic and has the potential to transform B cells. However, indolent EBV-positive B-cell neoplasms, such as MALT lymphoma, have rarely been reported. Different clinical settings can lead to the development of EBV-positive MALT lymphoma. EBV-positive MALT lymphomas occur in patients as a consequence of iatrogenic immune suppression following renal, cardiac, or liver transplant and were usually located in skin or subcutaneous tissue and, less frequently, in the stomach [11, 12]. In immunocompetent hosts, sporadic cases of EBV-positive MALT lymphomas with differing localization are described. In 1995, Liu *et al.* first described 2 cases of primary gastric EBV-positive MALT lymphoma out of 16 immunocompetent patients [13]. Subsequently, Oka *et al*. reported a case of EBV-positive MALT lymphoma of the stomach and a pulmonary EBV-positive Hodgkin lymphoma [14]. Daibata *et al*. studied an association of the human herpesviruses in a primary ocular MALT lymphoma and detected EBV in 4 of the 14 cases [15]. Interestingly, Shimakage *et al.* identified EBV in all five cases of pulmonary MALT lymphoma [16]. Although the data are scarce, one can speculate the dependence of EBV detection rate (and possible pathogenic role) on the location of MALT lymphoma in immunocompetent patients. In patients with autoimmune diseases, only six cases of EBV-positive MALT lymphoma have been described (Table 1). The lymphoma was in the thymus, lung, and mediastinal lymph nodes in one case [18]; in the stomach [17] and periparotid soft tissue in one case each [7]; and in the parotid gland in three cases [6, 7].

**Table 1:** Summary of previously reported cases of Epstein-Barr virus-positive mucosa-associated lymphoid tissue lymphoma in patients with autoimmune disease

Reference	Age (years)/sex	AD	Anatomic site	EBERs ISH	EBV PCR	LMP1	Treated with methotrexate
[17]	74/F	DM	Stomach	< 30% cells	NS	NS	No
[6]	NS	SS	PG	< 5% cells	+	–	NS
	NS	SS	PG	–	+	–	NS
[18]	49/F	SS	Thymus, lung, mediastinal LN	+/−	NS	NS	No
[7]	63/F	RA + SS	Periparotid soft tissue	+	NS	–	Yes
	54/F	RA + SS	PG	+	NS	–	Yes

−, a few scattered positive tumor cells; +, majority of the tumor cells were positive

*Abbreviations: DM* dermatomyositis, *SS* Sjögren’s syndrome, *RA* rheumatoid arthritis, *PG* parotid gland, *LN* lymph node, *AD* autoimmune disease, *NS* not specified, *EBV* Epstein-Barr virus, *EBERs* encoded small ribonucleic acids, *ISH in situ* hybridization, *PCR* polymerase chain reaction, *LMP1* latent membrane protein 1

Several studies have focused on the role of EBV in the pathogenesis of salivary gland MALT lymphoma. Diss *et al.* revealed EBV DNA in 3 of 36 cases of MALT lymphomas of the salivary glands. However, in a single EBV DNA-positive case, EBERs were found in less than 5% of tumor cells. In this regard, the researchers concluded that EBV is not associated with pathogenesis of MALT lymphomas of the salivary gland [19]. Royer *et al*. also did not find EBV DNA and EBERs in three cases of MALT lymphoma of the parotid gland in patients with SS [20]. Currently, six instances of EBV-positive MALT lymphoma of the salivary glands, including our patient’s case, have been described [6–8]. Two of these patients had congenital immunodeficiencies, and four had an autoimmune disease. All four patients with autoimmune pathology (including our patient) were diagnosed with primary SS or with SS associated with RA. SS is known to be a risk factor for salivary gland MALT lymphoma development [21], and the role of EBV in the pathogenesis of MALT lymphoma of the salivary gland in patients with impaired immunity may be underestimated.

Discrepancies in the assessment of EBV incidence in MALT lymphoma could be a result of methods used for EBV detection. EBERs, unlike EBV DNA, are expressed abundantly during latent EBV infection of B cells. Detection of EBERs by ISH is an accepted method of identifying latent EBV infection. For example, DLBCL is considered EBV-positive if over 80% of the tumor cells contain EBERs [22]. However, the percentage of EBER-positive cells that would result in MALT lymphoma to be considered EBV-positive has not been standardized. Several studies have employed PCR or Southern blots for EBV detection; however, these techniques cannot be employed to determine the prevalence and distribution of EBV-positive cells. Therefore, it is difficult to determine in many reported cases whether the MALT lymphoma was truly EBV-associated or whether the reported EBV-positive status represented unrelated latent EBV infection.

The EBV-positive MALT lymphoma in our study showed EBER positivity in numerous atypical B cells forming lymphoepithelial lesions within the salivary gland ducts; however, the tumor cells were LMP1-negative. This staining pattern suggests that those cells in the MALT lymphoma had a pattern of EBV latency type 0/I (EBER-positive, LMP1-negative).

The histological findings of the simultaneous occurrence of MALT lymphoma and DLBCL in the parotid gland led us to initially assume a clonal relationship between DLBCL and the MALT lymphoma and histological transformation of MALT lymphoma as the origin of the DLBCL. HGAL, Bcl-6, and MUM1 expression was upregulated in DLBCL compared with MALT lymphoma. The development of Bcl-6 and MUM1 expression in DLBCL is described in histological transformation of MALT lymphoma [23] and does not contradict our original assumption. However, the absence of EBER-ISH and EBV DNA in DLBCL in our patient promotes the notion that the DLBCL was not clonally related to the MALT lymphoma. The differing patterns of clonal rearrangement of the IGH gene between the MALT lymphoma and DLBCL served to confirm this assumption.

Patients treated with MTX have been shown to develop LPDs that share characteristics with lymphomas occurring in immunosuppression; the EBV genome has often been present in lymphoma cells, and spontaneous regression was possible after withdrawal of the drug alone. Most cases of MTX-associated LPDs have been DLBCL [24–26]. MTX-associated MALT lymphomas are very rare. Mariette *et al.* described three cases of MALT lymphoma associated with low-dose MTX in patients with RA without SS; these occurred in the stomach, lung, and the skin and orbit, respectively [24]. Kobayashi *et al*. reported a case of orbital MALT lymphoma that disappeared after MTX withdrawal in a patient treated with MTX for RA [27]. Unlike our patient’s case, ISH did not detect EBV in any of these MALT lymphomas. Gong *et al*. noted one case of EBV-positive MALT lymphoma in patients with SS and RA receiving MTX, etanercept, and infliximab immunosuppressive therapy. Discontinued immunosuppression and subsequent rituximab therapy led to clinical improvement with the resolution of parotid symptoms [7]. In our patient’s case, MTX was abolished 1 year before the increase in the left parotid gland. Therefore, a role of MTX in the development of lymphoma seems unlikely.

Little information is available on the relationship between leflunomide therapy and lymphoma development. However, Landais *et al.* checked VigiBase, a World Health Organization global database of individual case safety reports, and found 82 cases of lymphoma in patients treated with leflunomide [28]. Considering that our patient was taking leflunomide for 6 years before the diagnosis of lymphoma, we cannot rule out its effect on the development of lymphoma.

## Conclusions

We believe this case study highlights the importance of EBV screening of low-grade B-cell lymphoma in patient populations with autoimmune diseases. The role of EBV in the pathogenesis of MALT lymphomas may be underestimated in these patients. In addition, we believe that judging whether DLBCL is a result of low-grade B-cell lymphoma progression through histological transformation should instead be based on determining exact clonal relationships, even in cases that may seem morphologically obvious.

## Data Availability

All data generated or analyzed during this study are included in this report. The datasets used and/or analyzed during the current study are available from the corresponding author on reasonable request.

## Abbreviations

DLBCL: Diffuse large B-cell lymphoma
EBER: Epstein-Barr virus encoded small ribonucleic acid
EBV: Epstein-Barr virus
FDC: Follicular dendritic cell
FDG: Fluorine-18 fluorodeoxyglucose
IGH: Immunoglobulin heavy chain
ISH: *In situ* hybridization
LPD: Lymphoproliferative disorder
MALT: Mucosa-associated lymphoid tissue
MTX: Methotrexate
PCR: Polymerase chain reaction
RA: Rheumatoid arthritis
SS: Sjögren’s syndrome
